# Strategies used by professional rugby union clubs to manage players for artificial turf exposure

**DOI:** 10.17159/2078-516X/2020/v32i1a8276

**Published:** 2020-01-01

**Authors:** C D McKay, M J Cross, S P T Kemp, K A Stokes

**Affiliations:** 1 Department for Health, University of Bath, Claverton Down, Bath, BA2 7AY, UK; 2 Premiership Rugby, Regal House, 70 London Road, Twickenham, TW1 3QS, UK; 3 Rugby Football Union, Rugby House, Twickenham Stadium, 200 Whitton Road, Twickenham, TW2 7BA, UK; 4 Faculty of Epidemiology and Population Health, London School of Hygiene and Tropical Medicine, Keppel Street, London, WC1E 7HT, UK

**Keywords:** rugby, football wounds, injuries, risk factors, environment, decision-making

## Abstract

**Background:**

The use of artificial turf on rugby pitches is increasingly commonplace but there is limited evidence around its effects on injury, recovery, and performance. It is unclear whether this uncertainty influences player management strategies in professional clubs.

**Objectives:**

To understand how professional rugby union clubs in England approach player management for artificial turf, to explore how the beliefs of medical and strength/conditioning staff influence these decisions, and to determine whether differences exist between clubs with different levels of exposure to artificial surfaces.

**Methods:**

The study was a cross-sectional mixed methods study. Twenty-three medical and strength/conditioning staff members from 12 English Premiership Rugby Union clubs completed two bespoke questionnaires and participated in a semi-structured interview.

**Results:**

Two-thirds of the participants described formal club-level approaches to artificial turf. All participants from low-exposure clubs (<50% training and match time on artificial pitches) reported adjusting player recovery strategies following games on artificial turf to mitigate elevated muscle soreness and fatigue. Clubs with artificial surfaces at their home venues were less likely to adapt recovery than clubs with natural turf pitches. Regardless of exposure participants believed switching between surface types was a risk factor for injury. Medics reported that acute injuries associated with artificial turf exposure typically occurred at the foot or ankle, whereas abrasions and overuse injuries were more common and often affected the knees, hips and lower back. Players with compromised joints were less likely to be selected for matches on artificial surfaces.

**Conclusion:**

Player management around artificial turf is a focus for staff at professional rugby union clubs. Club practices vary by exposure and may consequently influence injury risk estimates.

Artificial turf is increasingly being used on rugby union pitches, both for training and competition. Yet, despite growing popularity and governing body regulations on minimum pitch standards, there is concern about its potential to increase injury risk.

Two systematic reviews examining injury risk across football codes (football, American football, rugby union) found comparable injury rates between turf types, aside from a slightly increased risk of anterior cruciate ligament (ACL) injury on artificial turf in American football^[1,2]^. Although one study in football has suggested that teams regularly training on artificial turf have higher rates of acute training and overuse injury than clubs with natural grass home pitches^[3]^, shifting between these two different surface types appears to have no effect on injury rates in this population^[4]^.

In rugby union, Fuller et al.^[5]^ examined match injuries in six elite men’s teams in Hong Kong over two seasons and reported no differences in incidence between surface types aside from a small, but not statistically significant increase in ACL injury risk (n=6; rate ratio=3.82; p=0.222). Another study comparing single season injury characteristics between two professional teams, one with World Rugby third generation certified artificial turf and the other with natural grass, found no difference in acute injury incidence between surfaces; however, overuse injuries appeared more likely on the artificial pitch during matches and in training^[6]^. Williams et al.^[7]^ conducted a small prospective cohort study in the highest level of professional rugby in England (Premiership) and, in a sample based on a single pitch, found no clear differences in injury incidence, severity, or burden between surface types. Notably, a prospective cohort study of two professional clubs (n=157 players) over three seasons demonstrated no overall difference in injury risk between grass (81.9 injuries/1000 match-hours, 95% CI: 72.2–92.5) and artificial surfaces (80.2 injuries/1000 match-hours, 95% CI: 69.9–91.7), but distinct injury patterns emerged (e.g. greater risk of foot injury on artificial turf)^[8]^. These findings are partially supported by ongoing injury surveillance across the Premiership, where two of 12 clubs currently have artificial surfaces at their match venues. This long-term surveillance programme has shown that injury severity, and consequently burden, may be increased on artificial surfaces (5 year burden = 3015 days absence/1000 match-hours, 95% CI: 2768–3285) when compared to grass (2433 days absence/1000 match-hours, 95% CI: 2342–2527), largely driven by more severe hamstring, foot and toe injuries^[9]^. These results align with anecdotal accounts from the rugby community, where there are persistent concerns that artificial surfaces may be riskier^[10]^. Altogether, this conflicting evidence base suggests that either artificial turf does not significantly increase injury incidence and there are misconceptions about its safety, or that players are being proactively managed to minimise their risk exposure, therefore affecting injury risk estimates.

There is some evidence that coaches may have more positive opinions of safety on artificial turf than athletes^[11]^, which likely has implications for player exposure to these surfaces during training and competition. In elite sport, medical and strength and conditioning (S&C) staff contribute significantly to these decisions as well but their beliefs and how greatly they influence practice are largely unknown. Therefore, the primary purpose of this study was to understand how professional rugby union clubs approach player management for artificial turf. Secondary objectives were to explore how the beliefs of medical and S&C staff influence these decisions, and to determine whether differences exist between clubs with different levels of exposure to artificial surfaces.

## Methods

This was a pragmatic, cross-sectional mixed methods study conducted from December 2016 – April 2017. It followed a concurrent triangulation strategy whereby quantitative and qualitative data were collected at the same time to permit comparison between the results obtained from each element^[13]^. This approach was selected to promote credibility by producing substantiated findings, and to shorten the data collection period within a congested professional sport setting. Data collection occurred during a site visit to each of the 12 English Premiership Rugby clubs, during which eligible participants provided consent prior to completing two study questionnaires and a semi-structured interview. Ethical approval was granted by the Research Ethics Approval Committee for Health at the University of Bath (EP 15/16 255).

Heads of Medical Services and Heads Strength & Conditioning (S&C) coaches from each club were purposively targeted for recruitment as they would be best placed to comment on club-level approaches to player management; however, to promote equitable opportunity to take part in the research, all staff in the Medical Services and S&C departments were invited to participate. Eligibility criteria were: (1) a member of the Medical or S&C Department at a Premiership club; (2) directly involved in player management; (3) conversant in English. Clubs were contacted directly via email and/or telephone (author MC) to initiate recruitment.

A 12-item demographic questionnaire was used to collect participant characteristics (i.e. club role and professional experience level). A 15-item ‘General Practices’ questionnaire captured current club practices and practitioner beliefs about injury risk on artificial turf using tick boxes and 7-point Likert scales. Both questionnaires were created for this study (author CM) based on instruments used to capture behaviours and beliefs in previous sports research^[12]^. These were face validated by the authorship team prior to use (supplementary online content).

One researcher (CM) developed an interview topic guide to steer the interview dialogue, whilst allowing participants to say as much as they wished (supplementary content). This focused on practices related to player management in the week leading up to, during, and in the week following a match played on artificial turf, and reflections on how/why these practices differed from those employed for natural grass. Interviews were conducted one-on-one for participants from 10 clubs. For the other two clubs, changes to the team’s schedule for the day meant that the Heads of Medical Services and S&C had to be interviewed together (e.g. two-on-one). Interviews were audio recorded for transcription and lasted 15–35 minutes.

Quantitative analysis was conducted using STATA [StataCorp Version 13, 2013]. Artificial turf exposure was arbitrarily dichotomised to preserve club confidentiality: clubs were classified as ‘low exposure’ if they spent less than 50% of their combined training and match time on artificial turf, or ‘high exposure’ if ≥50%. Differences in questionnaire responses between respondents from high- versus low-exposure clubs were assessed descriptively, given the exploratory nature of the research questions and the limited validation of the questionnaires.

A single investigator (CM), who is an experienced sport injury researcher but has no background in rugby union or any personal relationships with the clubs participating in the study, transcribed interview recordings verbatim and led the analysis process. Participants reviewed their transcripts to ensure accuracy (e.g. verification) before data were organised in NVivo [QSR International Pty Ltd. Version 11, 2015]. Thematic analysis followed the steps outlined by Braun and Clarke^[14]^: transcripts were read several times for familiarisation and inductive semantic coding was used to identify patterns in the data. Higher order themes were developed iteratively, following a recursive process of reviewing and defining emerging concepts^[13]^. To enhance rigour and trustworthiness, a second researcher (MC) independently coded 10% of the data to facilitate comparisons between coders, and all themes were reviewed for coherence.

Quantitative and qualitative data were integrated at the point of interpretation, allowing the authors to note areas of convergence within the findings to strengthen the knowledge claims of the study whilst exploring any lack of convergence that emerged (e.g. triangulation)^[13]^.

## Results

### Questionnaire outcomes

Participant characteristics are presented in Table 1. All 12 Premiership clubs were represented, including 13 medical staff (12 Heads of Medical Services and one match-day doctor) and 10 Head S&C coaches. Eighteen questionnaires were completed (78%), representing 11 clubs. Two Head S&C coaches declined participation because of competing time commitments, and five questionnaires were not returned for undisclosed reasons.

**Fig. 1:**
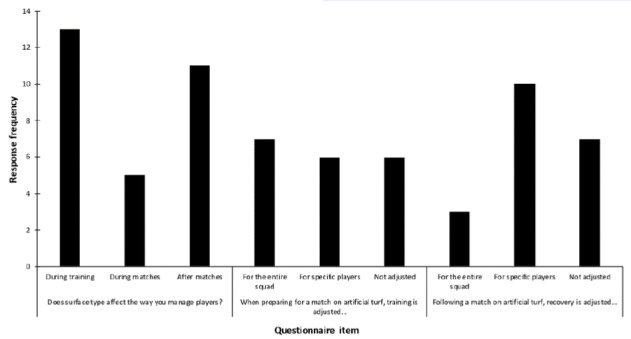
Number of respondents reporting specific current practices with respect to artificial turf

**Fig. 2:**
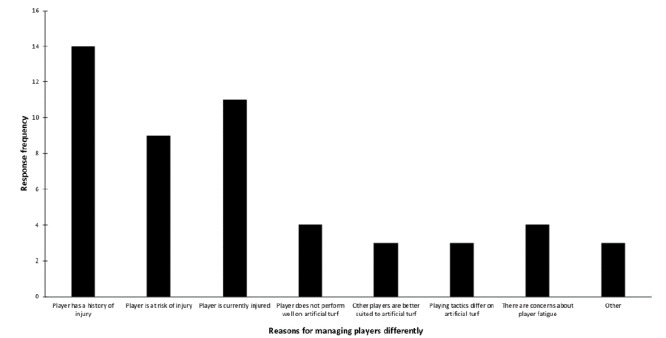
Number of respondents endorsing reasons players might be managed differently with respect to artificial turf

Three clubs had artificial match pitches and 10 clubs had regular access to artificial training pitches. Eleven respondents (61%) reported club-level plans for managing players for artificial turf. Six (33%) indicated that surface type influenced player selection for matches at their club. Participants from high-exposure clubs did not report managing players any differently during training (3/6 respondents) than those from low-exposure clubs (10/12 respondents). They were less likely to modify player management during match play (0/6 vs. 5/12), or to adjust recovery following artificial turf exposure (2/6 vs. 9/12). Questionnaire responses are summarised in Figures 1 and 2.

Twelve participants (67%) thought clubs with regular artificial turf exposure had a competitive advantage, but only for matches played on artificial surfaces. Most participants believed that, compared to natural grass, injury risk on artificial turf was slightly higher (median score 5/7, range 3–7). Personal concern about managing players for artificial turf varied widely (median score 5/7, range 1–7).

### Interview results

From the interviews, three higher order themes emerged: (1) perceptions of surface qualities and characteristics, (2) player interactions with artificial turf, and (3) player management approaches for artificial turf. Each theme comprised several sub-themes that reflected varied experiences across clubs and the professional opinions of individual participants (Table 2; full thematic tree in supplementary content). In the first theme, perceptions of surface qualities and characteristics, participants shared their positive and negative opinions about various features of artificial turf and discussed its benefits and drawbacks in the professional rugby union context. In particular, they spoke about the polarising nature of artificial turf and concerns they had about switching between surface types in terms of injury risk and competitive (dis)advantages. Theme two was player interactions with artificial turf, which highlighted athlete perceptions and preferences regarding surface types, and how these can influence complex individual and club-level decision-making around issues, such as player selection. Finally, the theme of player management approaches for artificial turf included participants’ explanations of how surface type affects game preparation and recovery practices as part of their day-to-day roles at their clubs. This theme brought to light how uncertainties about training adaptations and injury risk can create challenges in daily decision-making for practitioners.

### Perceptions of surface qualities

In terms of perceptions of surface qualities and characteristics, opinions about artificial turf were polarised, ranging from extremely positive to extremely negative. Those from clubs with artificial turf home match venues were complimentary, as were participants from some other clubs that used artificial turf training pitches. They cited the turf’s resilience to poor weather conditions, consistency when practicing technical skills, and cleanliness compared to grass pitches. Participants also identified the potential for faster gameplay:

*‘From the* *GPS data we tend to see higher max velocity speeds… we do find that the metres/minute goes up.’*

Those who disliked artificial turf primarily raised concerns over increased injury risk, particularly when transitioning between surface types:

*‘It’s not great, in my opinion, to be going from soft pitch to hard pitch, soft pitch to hard pitch. That’s when we tend to find we get guys pulling up with tight Achilles, tight hamstrings, groin tightness...’*

More than half of the participants (including those with and without artificial turf home venues) thought high-exposure clubs held a competitive advantage due to tactical experience and player adaptation to the training stimulus of the pitch. The remaining participants thought there was no advantage, indicating that grass pitches offered a challenge to clubs accustomed to artificial surfaces:

*‘They play on artificial turf every other week so they have an advantage in terms of being used to the way the ball bounces, the feel of the field, 50% of the time. But they also have a disadvantage 50% of the time when they go away and play on other surfaces.’*

### Player interactions with artificial turf

With respect to player interactions with artificial turf, participants said that players tend to either love it or hate it. Staff from high-exposure clubs suggested that after some initial hesitation, most players had grown to enjoy artificial turf because of an increased speed of play, greater surface consistency, and the cleanliness of the field. Low-exposure clubs reported more variability in player perceptions, particularly amongst those with a history of injury:

*‘If you’ve previously been hurt on an artificial pitch, then you blame the artificial pitch… You don’t hear anyone going, ‘I’ve been injured on grass, I don’t want to play on grass ever again, I’m just going to play on an artificial pitch.’ But you hear people that get hurt on an artificial pitch, and they’re straightaway going, ‘it’s the pitch, it’s the pitch.’*

There was also discussion about players who had been advised not to play on artificial turf for medical reasons, with some participants indicating that this could affect player selection:

*‘So, if we’re looking to sign somebody [and we have] an [artificial] training facility… I mean, if this player cannot train on that surface, and we’re saying he definitively cannot, then he cannot be here. So it does start to influence who you recruit into your organization.’*

### Player management approaches

When discussing player management approaches for artificial turf, several participants referenced formal management plans at their club, although these were more common amongst low-exposure clubs. They typically involved aspects of adjusted training, but the most common concern to emerge was uncertainty over periodisation and whether match preparation needed to be changed. Amongst low-exposure clubs, half elected to train on artificial turf leading into games on that surface, whilst the others continued training on grass:

*‘Do you accumulate familiarity on it, or do you do nothing so you’re not accumulating work and just take your hit at the weekend? I’m not sure if we’re doing the right thing or not.’*

Most clubs adjusted recovery protocols following games on artificial pitches. Several relied on markers of fatigue and training load to guide the extent of that adjustment on a per player basis alongside general squad-level training reductions up to 48 hours postgame. Participants identified increased muscle soreness and fatigue as the chief complaints associated with artificial turf. These were attributed either to unfavourable interactions between studded footwear and the turf, increased ground reaction forces or the increased speed and intensity of gameplay.

High-exposure clubs indicated that following an initial adaptation period, players no longer reported soreness or fatigue related to the turf. Some clubs reported that training on soft, wet pitches resulted in more muscle soreness and fatigue, which was alleviated by training on artificial pitches. Many participants wondered whether there is a strong relationship between artificial turf and injury. Around half of them believed that artificial surfaces do contribute to injury risk, although some thought this was primarily applicable to players with a history of injury:

*‘So we think, with a certain player group, we’ve got a good correlation that if we expose them to artificial turf, training or playing, that the likelihood of them getting injured is quite high.’*

Participants reported turf-related injuries including abrasions, ankle injuries (particularly syndesmosis sprains), tendinopathy, and lumbopelvic pain, though artificial turf was most commonly linked with overuse injuries. Concern was expressed for players with a history of soft tissue injury, lower extremity tendinopathy, or joint compromise (e.g. previous injury resulting in a reduced capacity to accommodate training and playing load). Most clubs took precautions against exposing affected players to high loads on artificial surfaces and often incorporated more intense recovery periods following exposure.

Considerable discussion focused on managing injured players through rehabilitation. With allowances for injury type, some medical staff favoured performing rehabilitation on artificial turf, believing it provides a consistent, clean surface for safety and re-acclimatises players to high training loads. Others routinely avoided artificial turf exposure because of a perceived increase in re-injury risk. Surface type also had the potential to influence return-to-sport decisions:

*‘I have made clear recommendation to delay a return-to-play of a recovering athlete because of the surface they were returning to.’*

Overall, the interviews highlighted several challenges around developing management strategies, including tactical aspects (i.e. understanding how surface properties affect ball behaviour) and logistical issues (i.e. accessing artificial surfaces for training). Participants spoke about how these concerns presented challenges to their daily decision-making, the most salient of which was balancing player welfare and performance within a context of competing priorities:

*‘…at the end of the day, they are there to do a job, so we’ve still got to protect them from a medical point of view but, you know, we want to give the player the best opportunity to get on the field.’*

Participants also identified four areas where additional research was needed to inform their practice: periodising training on artificial turf, injury risk, long-term player health, and appropriate footwear choices for artificial surfaces.

## Discussion

This study is the first to show that surface type is sufficiently influential to warrant pre-planned player management strategies in Premiership Rugby, but most clubs had formal approaches based on medical and coach experience rather than evidence.

The common belief was that switching between surfaces caused more fatigue and injury problems than consistently training on one surface type; however, not all reported practices aligned with this belief. High-exposure clubs trained predominantly on artificial turf because they believed it minimised training load changes and provided necessary environmental consistency for tactical development. It is unclear whether this provided physiological adaptations to artificial turf exposure, but staff at these clubs had lower perceptions of turf-related injury risk than their low-exposure counterparts and reported that players at their clubs made fewer fatigue-related complaints. Conversely, half of the low-exposure clubs trained on artificial pitches prior to games on that surface (to gain tactical familiarity) and the other half trained on grass (to minimise fatigue) in the preceding week. Both of these approaches enforce surface switching either in the week leading into or during a match, which is incongruent with reported injury risk concerns. Notably, these participants all expressed uncertainty about which approach was best and identified this as a priority area for research.

All participants from low-exposure clubs reported adjusting recovery following matches on artificial turf to account for elevated muscle soreness and fatigue. A study in professional football (n=13) found that a one-off exercise bout on artificial turf did not induce greater fatigue or delay physical recovery compared to natural grass amongst those who regularly played on artificial surfaces^[15]^. Similarly, Fletcher et al.^16]^ found no difference in sustained muscle soreness between surface types in rugby league. These findings contradict evidence from England’s Premiership; however, where Williams et al.^[7]^ measured slightly but consistently elevated self-reported muscle soreness in the four days following matches played on artificial pitches. Although this study’s findings are based on complaints made to medics/coaches rather than measuring player perceptions directly, participants speculated that higher speeds of gameplay on artificial turf may account for the soreness that they observed in their teams. There is some evidence showing decreased initial acceleration contact times and shorter contact times during cutting manoeuvres on artificial surfaces compared to grass, which may be perceived as “faster gameplay”;^[10]^ however, further investigation into the success of altered recovery paradigms is warranted to determine whether these practices reduce perceived fatigue/soreness or indeed promote positive physiological adaptations.

Team medics reported that acute injuries associated with artificial turf exposure typically occurred at the foot or ankle, whereas abrasions and overuse injuries were more common and often affected the knees, hips and lower back. This is consistent with injury surveillance outcomes in this population^[8,9]^ but the interviews highlighted particular concerns around the management of players with a history of joint compromise or tendinopathy. Although research into human-surface interactions on various surface types is emerging, practitioners are largely reliant on experience and athletes’ self-reported symptoms to guide management strategies. Importantly, the interview responses confirmed that ‘high risk’ players are often prophylactically managed to reduce artificial turf exposure and this may influence subsequent injury risk estimates.

Participants also suggested that turf type could affect return-to-play decisions and player selection. Recently, a small study (n = 30) investigated athlete’s perceptions toward artificial turf and found that artificial turf has greater acceptability amongst professional rugby players than footballers^[10]^. The present study has confirmed that there are mixed perceptions amongst medical and S&C staff as well which, when combined with player beliefs about safety and performance, have the potential to affect artificial turf exposure. Players who are prevented from returning from injury onto an artificial surface (or refuse to) could record a week or more of additional time loss, depending on how ‘return-to-sport’ is defined and captured, leading to significantly overestimated injury severity and burden in large scale surveillance studies.

Overall, the mixed-methods design of the present study was a strength-based approach in providing insight into the rationale supporting current player management practices. However, due to the study’s cross-sectional nature, practice changes through the season were unaccounted for. Moreover, the questionnaires were not fully validated prior to use and practitioner beliefs about injury risk may therefore have been under- or over-reported due to response bias or measurement error. As the study was limited to medical and S&C staff, the results may not capture other club-level decisions that could affect injury risk and thus the authors’ understanding may be incomplete. This study’s participant sample is also unlikely to be representative of all rugby union clubs or individual practitioners working in professional rugby union, as these settings and roles are heterogeneous and constantly evolving. Yet, this has provided a first insight into the predominant concerns of support staff at professional rugby clubs with respect to player management for different surface types and provides direction for future research.

## Conclusion

Player management approaches with respect to artificial turf in English professional rugby union are widely varied and largely experiential. Most significantly, there is evidence that players are in some cases being proactively managed to minimise artificial turf exposure, therefore potentially affecting injury risk and severity estimates. This has meaningful implications for injury surveillance strategies, medical and performance programmes, and player welfare initiatives.

## Supplementary Information





## Figures and Tables

**Table 1:** Participant characteristics

Participant characteristics (n=23)	Median (Range)
Age (years)	36 (31–57)
Experience in current role (years)	4 (1–13)
Tenure at current club (years)	2 (1–5)
Previous clubs (total number)*	1 (0–5)
Employment in professional rugby (years)	8 (1–18)

* Includes all previous professional/international clubs where the participant was previously employed, including those based in the UK and abroad.

**Table 2:** List of themes and subthemes

Themes	Subthemes
Perceptions of surface qualities and characteristics	Artificial turf polarises people Transitioning between surface types is a problem Some pitches are maintained better than others It may (or may not) give a competitive advantage
Player interactions with artificial turf	Players love it or hate it Increased speed of play means better performance The surface type we train on might affect player selection
Player management approaches for artificial turf	Formal management plans at the club Match preparation may (or may not) be adjusted Recovery strategies may (or may not) be tailored Is there a relationship between artificial turf and injury? Challenges in daily decision-making
