# Supplementary material for: Strategies used by professional rugby union clubs to manage players for artificial turf exposure
Source: S Afr J Sports Med. 2020 Jan 1;32(1):v32i1a8276. doi: 10.17159/2078-516X/2020/v32i1a8276 (PMC9924615; doi:10.17159/2078-516X/2020/v32i1a8276)
Supplement: Supplementary file 1 [file 2078-516X-32-v32i1a8276-s001.pdf]

Participant Questionnaire

Thank you for agreeing to complete this questionnaire. It should take you approximately 5 minutes to complete. Please answer all questions as accurately as possible.

The information you provide will remain strictly confidential, will not be released to your club, Premiership Rugby or the Rugby Football Union, and will not jeopardize any future association with the University of Bath.

SECTION A: Personal Details

1. Current age (years): _______________

2. ☐ Male ☐ Female

3. What is your current professional role at your rugby club?

   ____

4. How long have you occupied this role at your current club (years)? ____________________

5.
   a. Have you previously performed this role at another professional rugby club?

      ☐ Yes

      ☐ No

   b. If yes, please identify the number of clubs at which you have performed this role: ______________

   c. If yes, how many of these were Premiership Rugby clubs in the UK? ______________

6. Altogether, how many years of experience do have…

    a. … performing your current role in professional rugby? _______________

    b. … being employed by a professional rugby club? _______________

    c. …playing rugby professionally? _______________

SECTION B: Club Details

7. Please list all of the training facilities your club has used in the past 6 months:

____

____

____

____

8. Which of these facilities is your club's regular training venue? ____

    a. Does it have artificial turf?

        ☐ Yes
        ☐ No

9. What percentage of the time do your players train on artificial turf (0-100%)? _______________

10. Does your club's home match facility have artificial turf?

    ☐ Yes
    ☐ No

11. During the Premiership season, do you regularly travel with the club to away matches?

    ☐ Yes
    ☐ No

12. How many players are currently in your first team squad? _______________

Player Management/Artificial Turf 2016

Thank you for agreeing to complete this questionnaire. It should take you approximately 10 minutes to complete. Please answer all questions thoughtfully and as accurately as possible. There are no right or wrong responses; we are merely interested in your personal views.

The information you provide will remain strictly confidential, will not be will not be released to your club, Premiership Rugby or the Rugby Football Union, and will not jeopardize any future association with the University of Bath.

SECTION A: Current practices

1. Does surface type (turf versus grass) affect the way you manage players…
   a. During training? ☐ Yes ☐ No
   b. During matches? ☐ Yes ☐ No
   c. After matches ☐ Yes ☐ No

2. When preparing for a match on artificial turf, which of the following applies at your club (check all that apply)?
   ☐ Training is adjusted for the entire squad
   ☐ Training is adjusted for specific players
   ☐ Training is not adjusted

3. Does surface type (turf versus grass) affect player selection for matches for your club?
   ☐ Yes ☐ No

4. When recovering from a match on artificial turf, which of the following applies at your club (check all that apply)?
   ☐ Recovery is adjusted for the entire squad
   ☐ Recovery is adjusted for specific players
   ☐ Recovery is not adjusted

5. What are the reasons you might manage a player differently for artificial turf (check all that apply)?

☐ Player has a history of injury

☐ Player is at risk of injury

☐ Player is currently injured

☐ Player does not perform well on artificial turf

☐ Other players on the squad are better suited to playing on artificial turf

☐ Playing tactics/strategies differ on artificial turf

☐ There are concerns for player fatigue

☐ Other (please describe: ____)

6. In your opinion, do clubs that regularly play on artificial turf manage players differently than clubs that do not regularly play on turf?

☐ Yes ☐ No

7. In your opinion, do clubs that regularly play on artificial turf have a competitive advantage over clubs that do not regularly play on turf?

☐ Only for matches played on artificial turf

☐ For all matches, regardless of surface type

☐ They do not have an advantage

8. Does your club have an organised plan for managing players on artificial turf?

☐ Yes ☐ No

9. Do you regularly evaluate how effective your strategies are for managing players on artificial turf?

☐ Yes ☐ No

10. What sources of information do you rely on when deciding how to manage players for artificial turf (check all that apply):

- ☐ Personal experience/training
- ☐ Advice from colleagues within the club
- ☐ Advice from colleagues outside of the club
- ☐ Club policies
- ☐ Professional organisations/colleges
- ☐ Scientific publications/evidence
- ☐ Internet media
- ☐ Print media (e.g., magazines)
- ☐ Other media
- ☐ Conference presentations
- ☐ Other (please describe: ____)

SECTION B: Personal perspectives

11. In your opinion, **overall injury risk** in professional rugby is:

	1	2	3	4	5	6	7	
**Low**	Extremely	Quite	Slightly	Neither	Slightly	Quite	Extremely	**High**

12. Compared to natural grass, **injury risk** on artificial turf is:

	1	2	3	4	5	6	7	
**Lower**	Extremely	Quite	Slightly	Neither	Slightly	Quite	Extremely	**Higher**

13. In your opinion, are some players' performance levels affected by artificial turf?

	1	2	3	4	5	6	7	
**Disagree**	Strongly	Somewhat	Slightly	Neither	Slightly	Somewhat	Strongly	**Agree**

14. Overall, how concerned are you about effectively managing players for artificial turf?

**Unconcerned**	1	2	3	4	5	6	7	**Concerned**
	Extremely	Quite	Slightly	Neither	Slightly	Quite	Extremely	

15. In what areas would you like to see more evidence to support your player management strategies (check all that apply?

- ☐ Player performance on turf
- ☐ Injury risk on turf
- ☐ Recovery from playing on turf
- ☐ Competitive tactics specifically for turf
- ☐ Effective training strategies for turf
- ☐ Injury prevention for turf
- ☐ Other (please describe: ____)
