# Supplementary figures and images for: Strategies used by professional rugby union clubs to manage players for artificial turf exposure
Source: S Afr J Sports Med. 2020 Jan 1;32(1):v32i1a8276. doi: 10.17159/2078-516X/2020/v32i1a8276 (PMC9924615; doi:10.17159/2078-516X/2020/v32i1a8276)

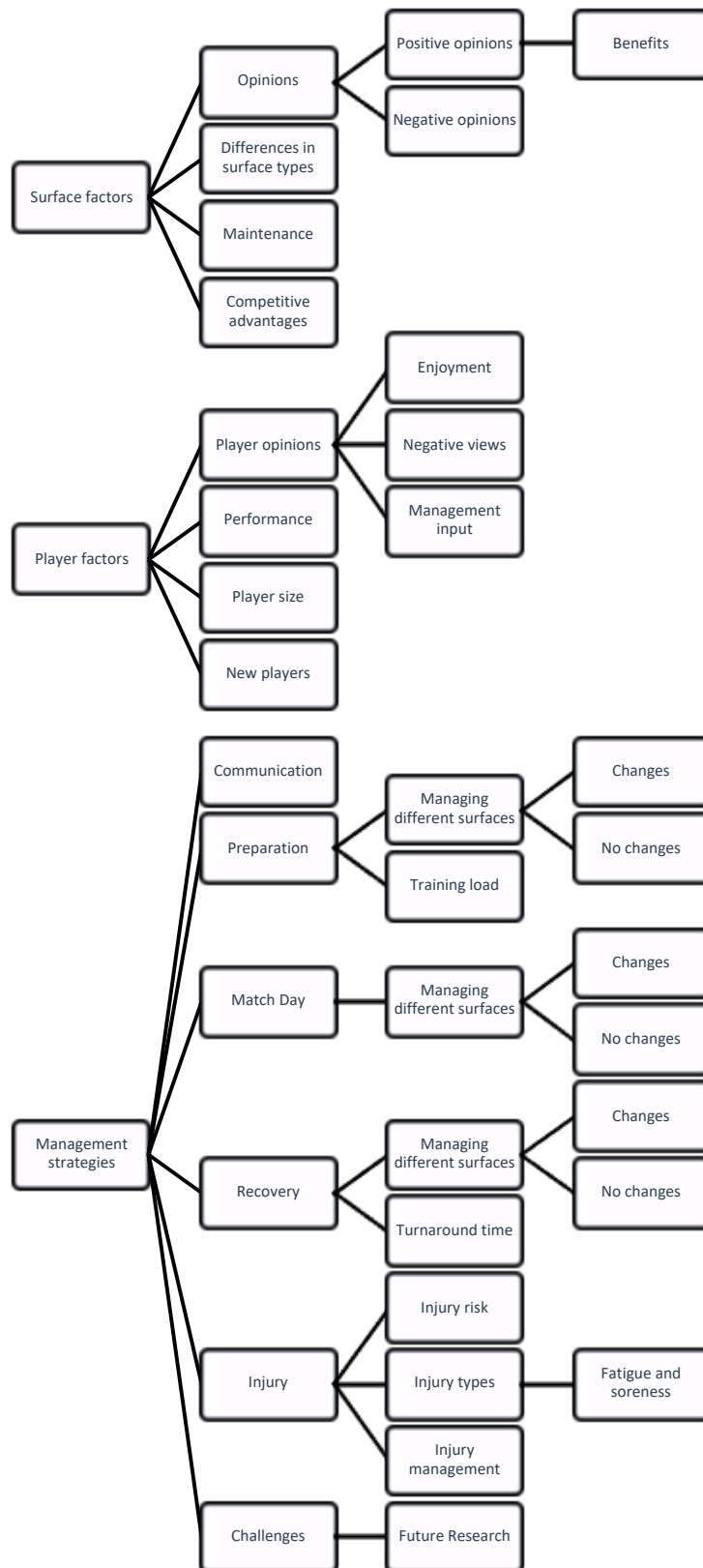

Supplement: Supplementary file 2 [file 2078-516X-32-v32i1a8276-s002.pdf]
